# Amino Acid Availability of a Dairy and Vegetable Protein Blend Compared to Single Casein, Whey, Soy, and Pea Proteins: A Double-Blind, Cross-Over Trial

**DOI:** 10.3390/nu11112613

**Published:** 2019-11-01

**Authors:** Jue Liu, Marianne Klebach, Monique Visser, Zandrie Hofman

**Affiliations:** Danone Nutricia Research, 3584 CT Utrecht, The Netherlands; marianne.klebach@danone.com (M.K.); Monique.Visser@danone.com (M.V.)

**Keywords:** protein quality, enteral tube feed, protein blend, vegetable protein, amino acid composition, non-essential amino acid, leucine, methionine, arginine, glycine

## Abstract

Protein quality is important for patients needing medical nutrition, especially those dependent on tube feeding. A blend of dairy and vegetable proteins (35% whey, 25% casein, 20% soy, 20% pea; P4) developed to obtain a more balanced amino acid profile with higher chemical scores, was compared to its constituent single proteins. Fourteen healthy elderly subjects received P4, whey, casein, soy, and pea (18 g/360 mL bolus) on five separate visits. Blood samples were collected at baseline until 240 min after intake. Amino acid availability was calculated using incremental maximal concentration (*i*C_max_) and area under the curve (*i*AUC). Availability for P4 as a sum of all amino acids was similar to casein (*i*C_max_ and *i*AUC) and whey (*i*C_max_) and higher vs. soy (*i*C_max_ and *i*AUC) and pea (*i*C_max_). Individual amino acid availability (*i*C_max_ and *i*AUC) showed different profiles reflecting the composition of the protein sources: availability of leucine and methionine was higher for P4 vs. soy and pea; availability of arginine was higher for P4 vs. casein and whey. Conclusions: The P4 amino acid profile was reflected in post-prandial plasma levels and may be regarded as more balanced compared to the constituent single proteins.

## 1. Introduction

Protein is a crucial macronutrient and is fundamental to maintaining human body function. The nutritional value of dietary protein sources depends primarily on their amino acid composition and bioavailability. An optimal dietary protein source may be defined by its ability to provide all amino acids required for optimal protein synthesis in muscles and other organs [1,2,3]. The quality of protein used in enteral tube feed products has, therefore, received much attention, especially for individuals depending on tube feeding as their sole source of nutrition [1,2,3].

The amino acid requirement during illness cannot be easily quantified. The World Health Organization, the Food and Agriculture Organization of the United Nations, and the United Nations University (WHO/FAO/UNU) protein report (2007) [4] for essential amino acids in healthy individuals is generally used as a reference to assess the quality of protein sources used in medical nutrition. Chemical scores can be calculated for each amino acid by comparing the amino acid profile of the protein to the recommended amino acid profile. The amino acid with the lowest chemical score is defined as the first limiting amino acid. It has been hypothesized that amino acids with chemical scores higher than the limiting amino acid cannot be efficiently utilized for protein synthesis [1,2,5]; therefore, the nutritional quality of a protein source can be determined by the chemical score of the first limiting amino acid.

Non-essential amino acids, which can be synthesized in the human body from other amino acids, have often been overlooked when assessing the quality of dietary proteins. Importantly, during illness, the body may not always synthesize sufficient amounts of non-essential amino acids required for optimal protein synthesis [1,6,7,8]. A previous study indicated that there can be inefficient metabolic conversion from essential to non-essential amino acids in patients with traumatic brain injury [8]. In addition, some amino acids (such as cysteine, arginine, glycine, and glutamine) may have unique roles in physiological functions beyond their role as substrates for protein synthesis [3,6,9,10,11]. Therefore, it can be hypothesized that certain non-essential amino acids can be seen as conditionally essential because they could become rate limiting for protein synthesis and other functions [1,3,7,12,13]. A dietary protein source providing an amino acid pattern with higher amounts of both limiting essential and conditionally essential amino acids can be considered as a protein with a more balanced amino acid pattern. This may prevent unnecessary metabolic amino acid transamination and thus support optimal protein synthesis and other functions. A more balanced amino acid provision may be particularly relevant for patients dependent on tube feeding as their only source of nutrition.

We propose to consider all individual amino acids in the determination of protein quality, as amino acids can be seen as individual nutrients with specific functions. The WHO/FAO/UNU report 2007 [4] introduced a recommended amount for cysteine; however, no official recommendations for non-essential amino acids exists. In a review paper by Wu, published in 2016, dietary requirements for all non-essential amino acids were proposed for healthy humans [9]. Although this is not an official recommendation and requires further validation, these data allow us to do an exploratory evaluation of the complete amino acid profile of dietary proteins.

Most commercially available enteral tube feed products contain a single type of protein. Dairy proteins (casein or whey) are normally used, because they contain, on average, higher amounts of essential amino acids than provided by vegetable proteins. However, both casein and whey have a lower content of certain non-essential amino acids. An exploratory calculation of chemical scores, based on the proposed non-essential amino acids requirements mentioned above, shows lower chemical scores for glycine and arginine as compared with those for vegetable proteins (Table 1). This could be interpreted as a less balanced non-essential amino acid profile. To create a more balanced amino acid profile, a protein blend consisting of 35% whey, 25% casein, 20% soy, and 20% pea (P4 protein) was developed, yielding an amino acid profile with a higher chemical score for both essential and non-essential amino acids compared to single protein sources (Table 1).

It is important to determine whether the more balanced amino acid profile (i.e., higher chemical scores) of the protein blend is reflected in the plasma, giving an indication of the systemic availability. Previous studies of milk or casein compared to soy proteins showed that the plasma amino acid pattern was a reflection of the amino acid content of the consumed protein, i.e., the amino acids that were present in higher (or lower) concentrations in the protein produced higher (or lower) plasma levels, respectively [14,15]. The aim of this study was to determine post-prandial amino acid availability following consumption of P4 protein compared to single protein sources. We specifically focused on four amino acids, leucine, methionine, arginine, and glycine, because they were identified as either the first or second limiting amino acids in the tested proteins (Table 1).

## 2. Materials and Methods 

### 2.1. Subjects

Eligible subjects were recruited from a healthy volunteer database, who were: (a) aged ≥ 65 years; (b) between 20 and 30 kg/m^2^ for body mass index; and (c) judged by the investigator to be in good health. Prior to participation in the study, we confirmed that the subjects were in a fasting state from 22:00 h the day before the study visits; were not using laxatives, antacids, anticonvulsants, prokinetics, or any medication influencing gastric acid production; were not taking nutritional supplements, with the exception of (multi) vitamin and/or mineral supplements; were not current smokers; had not consumed alcohol within the previous 24 h; and had not done any sort of intense physical activity 24 h prior to the study visits. All subjects were required to maintain normal dietary habits for the duration of the study. 

The study was registered in the Dutch Trial Register on 2 September, 2015 (registration number 5420) and it was approved by the Medical Ethical Review Committee of azM/UM, Maastricht, The Netherlands (number NL 52956.068.15/ METC 153027). The study was conducted according to the International Conference on Harmonization Guideline for Good Clinical Practice, in compliance with the ‘World Medical Association Declaration of Helsinki’ (64th WMA General Assembly, Fortaleza, Brazil, October 2013), and subject to the local laws and regulations of The Netherlands. All subjects provided written informed consent prior to study screening. 

### 2.2. Study Design 

**Figure 1:**
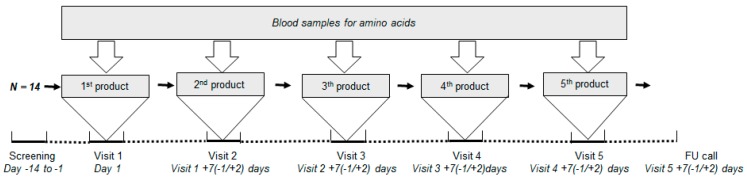
Schematic diagram of study design. FU = follow-up.

This study had a double-blind and cross-over design and was performed at R&D Group VitaK (Maastricht, The Netherlands). Subjects received all five study products sequentially, in a randomized order, on separate visits (Figure 1). The product sequence was generated by a computer random number generator. Investigators and subjects were blind to the allocated study products for the duration of the study.

Subjects attended the research center in a fasting state on five separate mornings, approximately one week apart (6 to 9 days after the preceding visit). An intravenous cannula was placed before the first (baseline) blood sample was taken. Subjects consumed a full dose of study product (volume of 360 mL) within 5 min and then rinsed their mouth with 30 mL of water. The study team recorded the actual start and stop times of study product intake. After consumption of the study product, venous blood samples (6 mL) were collected at 12 time-points over the next 4 h (15, 30, 45, 60, 75, 90, 105, 120, 150, 180, 210, and 240 min). At 125 min, the subjects drank 150 mL of water completely within 5 min. During the 4-h assessment period, subjects rested in a sitting or semi-recumbent position. Adverse events (AEs) and serious adverse events (SAEs) were recorded.

### 2.3. Study Products

Five products were tested: P4 protein blend (Nutricia, Utrecht, The Netherlands), casein, whey, soy, and pea. Table 1 shows the amino acid composition of the study products. The study products were food-graded produced by NIZO food research B.V, Ede, The Netherlands. The products were prepared by blending 18 g pure protein in 360 mL of water. No extra calories (i.e., fat, carbohydrate, or micronutrients) were added. Sucralose and flavors (i.e., caramel, orange, and peach) were used to improve the taste and palatability of the protein solutions. The protein solutions were sterilized and subsequently homogenized. The five products had a similar pH (around 7.1).

During the study day, the study products (18 g protein/360 mL) were prepared by dedicated unblinded site staff who were not otherwise involved in the study. The study products were prepared no more than 1 h before consumption and were served in blinded cups with a lid to maintain the double-blind design.

### 2.4. Blood Analysis 

Blood levels of 16 amino acids (histidine, isoleucine, leucine, lysine, methionine, phenylalanine, threonine, tryptophan, valine, alanine, arginine, aspartic acid, glutamic acid, glycine, serine, and tyrosine) were analyzed. Serum samples were stored at −20 °C until shipment to Danone Nutricia Research for analysis. After precipitation of proteins and polypeptides with perchloric acid, samples were centrifuged. The content of the individual amino acids was determined by ultra-fast liquid chromatography (Shimadzu Benelux) using a pre-column derivatization with ο-phthaldialdehyde and fluorimetry as detection. Genetic markers are explicitly excluded from future analysis. 

### 2.5. Outcome Parameters and Statistical Analysis

The parameter incremental maximal concentration (*i*C_max_) was defined as the maximum increase in plasma amino acid concentration observed post baseline in a subject during a study visit (from t = 15 min to t = 240 min). The parameter incremental area under the curve (*i*AUC) was calculated in the following equation:

$${}_{i}AUC_{0-4hr}=\overset{11}{\underset{i=0}{\sum }}\frac{\left[\left(C_{i+1}-baseline\right)+(C_{i}-baseline\right)]\left(t_{i+1}-t_{i}\right)}{2},$$

where i are the measurements taken at different time points after the start of the study product intake; $t_{i}\;\mathrm{and}\;t_{i+1}$ are actual time points relative to start of study product intake; $C_{i}\;\mathrm{and}\;C_{i+1}$ are the observed amino acid concentrations at time points and $t_{i}\;\mathrm{and}\;t_{i+1}$, and $C_{0}$ represents the baseline value. If the *i*AUC 0–4 h is a negative value, it is considered as zero.

Both parameters *i*C_max_ and *i*AUC were calculated for the sum of total amino acids (TAA) and for all individual amino acids. Statistical comparisons were performed for TAA and four key individual amino acids (leucine, methionine, arginine, and glycine). The parameters were transformed prior to statistical analysis using a logarithmic transformation (natural log). For all pairwise comparisons, a linear mixed effects model including period and study product as fixed effects, subject as random effect, and a compound symmetry covariance matrix with the Kenward-Roger option of degrees of freedom was performed. The assumption of homoscedasticity was checked for all parameters, and in the case a parameter showed strong evidence for variance heterogeneity, an unstructured correlations covariance matrix was used with the Kenward-Roger option of degrees of freedom. The assumption of normality of the residuals was determined based on a Q-Q plot and skewness and kurtosis statistics. In all analyses, statistical significance was tested using two-sided tests at the statistical significance level of α = 0.05. No corrections of multiple testing were performed due to the exploratory nature of the study. Analyses were performed based on the intention to treat (ITT) population including all randomized subjects. The statistical analysis was performed using SAS^®^ software (SAS Enterprise Guide 4.3 for Windows, SAS Institute Inc., Cary, NC, USA).

For each amino acid, three bar charts were created, i.e., mean of *i*C_max_, mean of *i*AUC, and amino acid content of the study proteins (expressed as g/100 g). Visual inspection of the bar charts was used as a pragmatic approach to explore the relationship between post-prandial individual amino acid profile and the amino acid content of the study proteins.

## 3. Results

### 3.1. Subjects 

Twenty individuals were screened, and 14 subjects were eligible to receive study products in a random sequence. Baseline characteristics are listed in Table 2. Thirteen subjects completed the study and 1 subject withdrew early because of difficulties with blood drawing during the first visit. All 13 subjects complied with the protocol for all study visits. Three adverse events (i.e., migraine, nasopharyngitis) were reported during the study period, but were not related to the study products after assessment by the investigator.

### 3.2. Post-Prandial Plasma Concentration of the Sum of TAA

**Figure 2:**
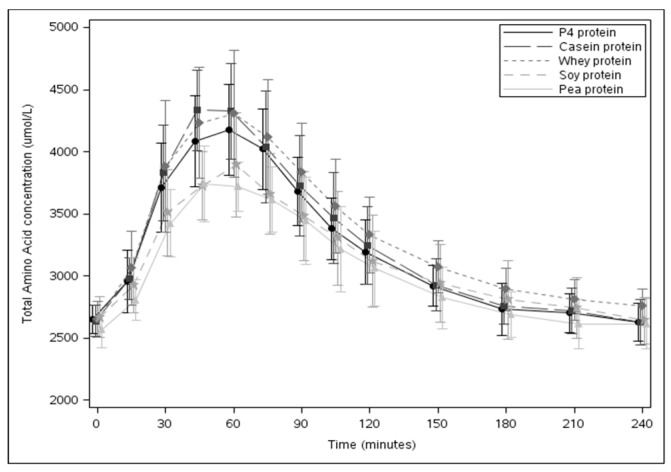
Post-prandial plasma TAA responses of the study products (mean ± SD).

Figure 2 shows changes in plasma concentration of TAA over time (mean ± SD). Table 3 shows plasma concentrations of TAA expressed as baseline concentration, *i*C_max_, and *i*AUC values for P4 protein vs. other study products. There were no statistically significant differences in the plasma TAA concentration at baseline between study products. Analysis of the post-prandial plasma TAA response with P4 protein showed no statistically significant differences from casein (*i*C_max_, *p* = 0.101; *i*AUC, *p* = 0.207) and whey protein (*i*C_max_, *p* = 0.249), whereas, a statistically significant higher response was seen with P4 protein compared to soy (*i*C_max_, p < 0.001; *i*AUC, *p* = 0.040) and pea protein (*i*C_max_, *p* = 0.001). 

### 3.3. Post-Prandial Plasma Concentration of Individual Amino Acids 

Table 4 shows post-prandial plasma concentrations (baseline, *i*C_max_, and *i*AUC) of leucine, methionine, arginine, and glycine for P4 protein vs. other study products. There were no statistically significant differences in the individual amino acid responses with P4 protein compared to casein for plasma leucine concentration (*i*C_max_, *p* = 0.051; *i*AUC, *p* = 0.612) and whey for plasma methionine concentration (*i*C_max_, *p* = 0.417; *i*AUC, *p* = 0.293). When compared to vegetable proteins, the responses with P4 protein were much higher for both plasma leucine and methionine concentrations; these differences were statistically significant (*i*C_max_ and *i*AUC, all *p*-values < 0.001).

A statistically significant higher plasma arginine concentration was observed with P4 protein compared to casein (*i*C_max_, *p* = 0.008; *i*AUC, *p* = 0.003) and whey proteins (*i*C_max_ and *i*AUC, both *p*-values < 0.001). Although glycine concentration with P4 protein was higher than with dairy proteins, the differences were not statistically significant. P4 protein resulted in a statistically significant lower arginine concentration than soy (*i*AUC, *p* = 0.002) and pea proteins (*i*C_max_, *p* < 0.001; *i*AUC, *p* = 0.002) and a statistically significant lower glycine concentration than soy protein (*i*AUC, *p* = 0.035).

**Figure 3:**
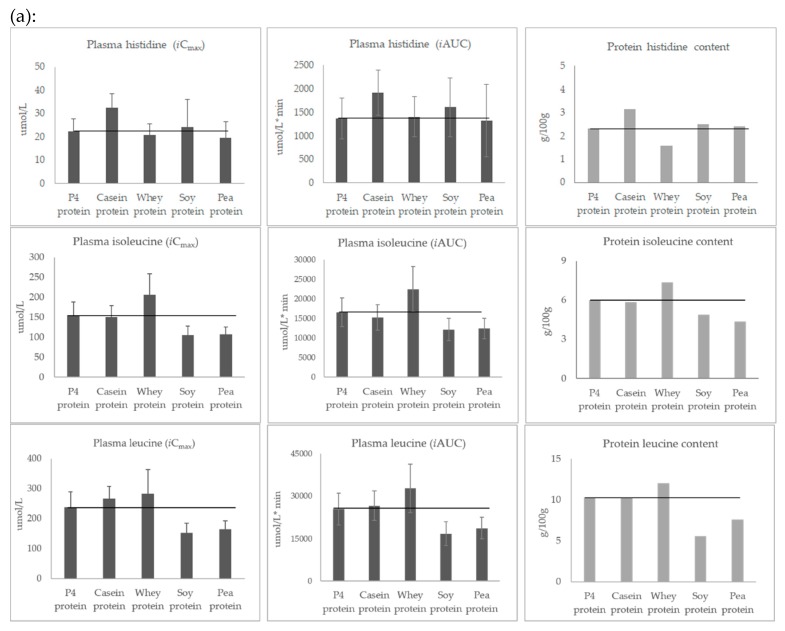

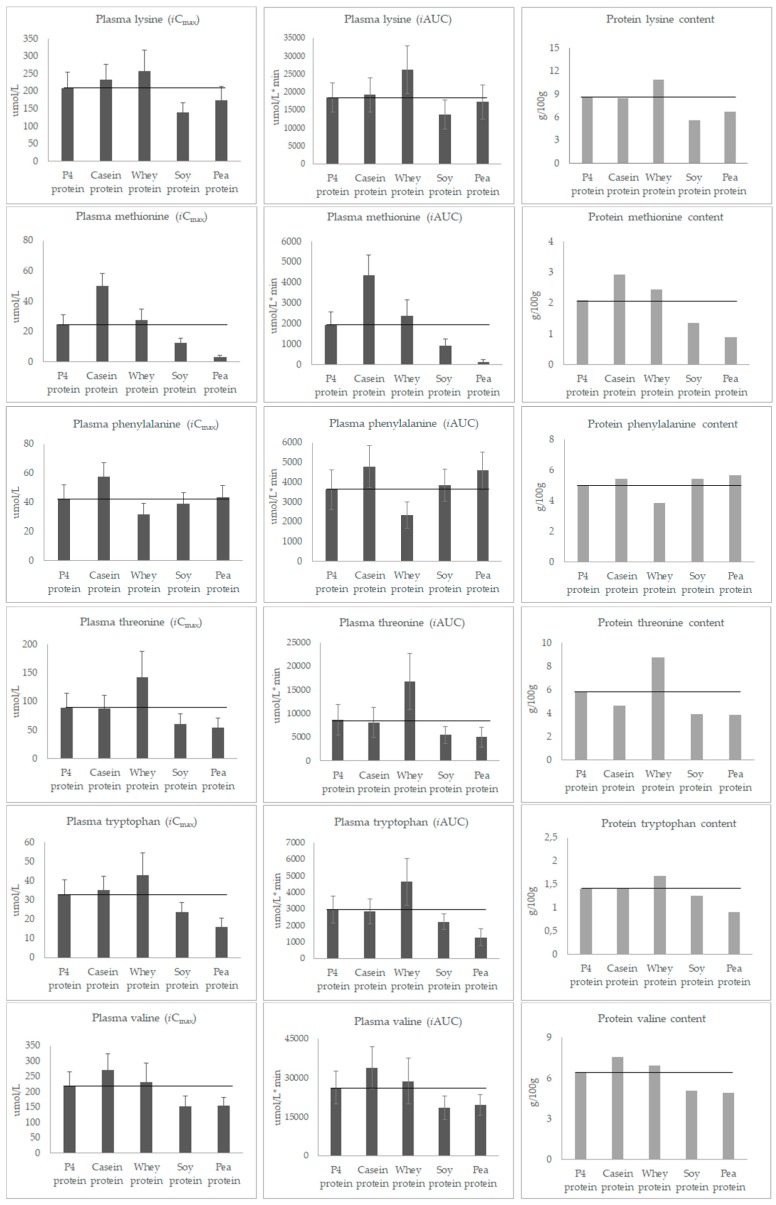

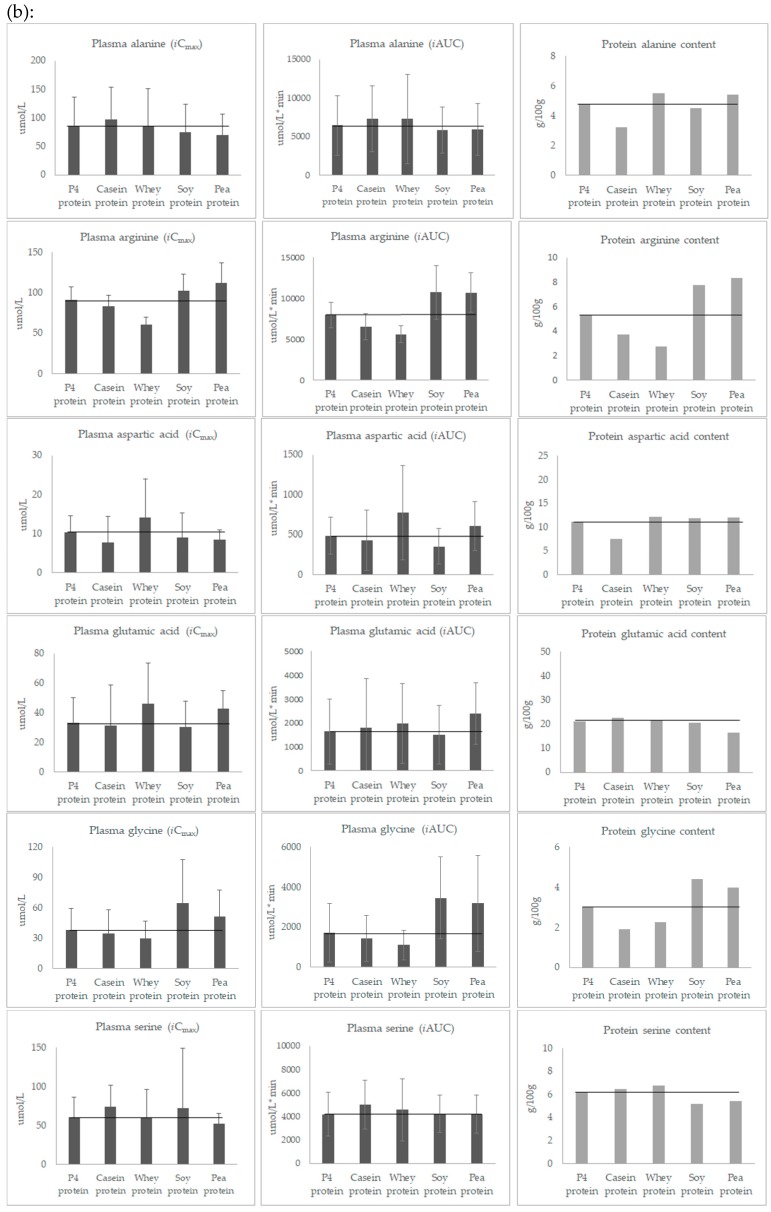

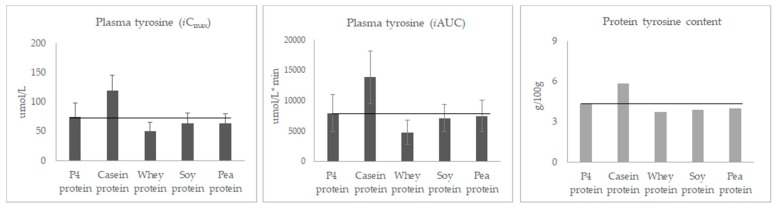
(**a**) Post-prandial plasma *i*C_max_ and *i*AUC for essential amino acids. (**b**) Post-prandial plasma *i*C_max_ and *i*AUC for non-essential amino acids (mean ± SD).

Figure 3 shows for each amino acid the post-prandial plasma levels (mean of *i*C_max_ and *i*AUC) and the amount of individual amino acid (g/100 g of protein) contained in the study products. Visual inspection of these bar charts indicates the amino acid profile pattern was similar for the two parameters, *i*C_max_ and *i*AUC. In addition, the post-prandial amino acid profiles were generally in line with the amino acid composition of the consumed protein sources—with three exceptions: alanine, aspartic acid, and glutamic acid. For example, results for *i*C_max_ and *i*AUC show that casein and pea protein, which contained the most and least methionine, resulted in the highest and lowest plasma methionine concentrations, respectively, while whey and soy protein, which contained the most and least leucine, resulted in the highest and lowest plasma leucine concentrations, respectively. 

On Figure 3, horizontal black lines have been added to facilitate visual comparison of responses (*i*C_max_ and *i*AUC) seen with P4 protein and other study products. For all amino acids, P4 protein resulted in a post-prandial response in the middle of the range of the post-prandial responses of casein, whey, soy, and pea proteins. For example, listing the protein sources in ascending order according to plasma levels of amino acids shows: soy and pea < P4 and casein < whey for leucine and lysine; whey < P4, soy and pea < casein for phenylalanine and tyrosine; and whey and casein < P4 < soy and pea for arginine. 

## 4. Discussion

In this cross-over study, the post-prandial amino acid availability after intake of a protein blend (P4 protein) was compared to its constituent single protein sources (i.e., casein, whey, soy, and pea) in healthy elderly subjects. Statistical analysis of the results showed that the post-prandial plasma response as a sum of all amino acids contained in P4 protein was similar to casein (*i*C_max_ and *i*AUC) and whey (*i*C_max_), and higher than soy (*i*C_max_ and *i*AUC) and pea (*i*C_max_). However, the same pattern was not observed for post-prandial individual amino acid responses. Based on the post-prandial availability of leucine, methionine, arginine, the responses with both dairy and vegetable proteins were either statistically significantly higher or lower than with P4 protein, respectively. More importantly, P4 protein consistently showed a post-prandial response in the middle of the range of responses seen with the other single protein sources. The post-prandial amino acid profiles reflected the amino acid content of the single protein sources. Taken together, our data suggest that the ingestion of P4 protein provides a more balanced post-prandial amino acid availability compared to single protein sources. In addition, our study highlights the importance of evaluating the amino acid availability of a dietary protein at an individual level, rather than as a sum of the total.

As shown in Figure 3, the protein sources used in this study differ largely in their amino acid composition, which in turn appears to directly influence the availability of amino acids in plasma (except for alanine, glutamic acid, and aspartic acid). This observation is consistent with previous studies [14,15], which also showed that the post-prandial individual amino acid response reflected the constituent amounts in the source products. While these previous studies compared milk or casein protein to soy protein, we tested more protein sources including casein, whey, soy, pea, and a mixture of all four, providing a comprehensive and comparative assessment of amino acid availability with P4 protein and its constituents. The absolute amount of amino acids in the circulating plasma over 4 h after intake was measured, knowing that the changes in plasma response are regulated by many factors, including digestion and absorption rates, the splanchnic area amino acid extraction, hormone secretion, protein synthesis and breakdown rates, and the level of de- and transamination [16]. Therefore, the results from this study do not give a specific indication of the digestibility of the tested protein sources, such as protein digestibility-corrected amino acid score (PDCAAS) or digestible indispensable amino acid score (DIAAS) [4,17]. The parameters *i*C_max_ and *i*AUC used in this study indicate systemic amino acid availability. Although *i*C_max_ and *i*AUC results were not always consistent with each other from a statistical perspective (e.g., the arginine response with P4 protein vs. soy was significant for *i*AUC, but not *i*C_max_), visual inspection of the bar charts showed they always had a similar post-prandial pattern. As shown in Figure 3, the lowest and highest amino acid content of the source proteins was reflected in the lowest and highest post-prandial responses, respectively, for both *i*C_max_ and *i*AUC. Thus, our data confirm the primary role of amino acid composition on post-prandial individual amino acid availability. 

The results of this study showed substantially greater availability of leucine and methionine with P4 protein than with vegetable proteins (e.g., *i*AUC of leucine and methionine were ~1.5 and 2 times higher with P4 protein vs. soy). Moreover, P4 protein resulted in a statistically significant increase in arginine availability and a non-significant increase in glycine availability compared to casein and whey (e.g., *i*AUC arginine and glycine were ~1.4 times higher with P4 protein vs. whey protein). These data indicate that intake of P4 protein can lead to a higher plasma concentration of the limiting amino acid in the protein source compared to the other single protein sources. This finding is relevant, because higher availability of limiting amino acids could increase the efficiency of other amino acids and thus improve protein accretion [1,5]. In this context, however, it is also important to note that a high intake of certain individual amino acids may not be beneficial and could even have a negative effect on protein synthesis [2]. For example, a recent cross-over study [18] found that native whey containing around 25% more leucine than regular whey protein did not further enhance muscle protein synthesis in subjects during the post exercise period. Paddon-Jones [19] also showed that provision of additional arginine in isolation does not appear to affect muscle protein synthesis effectively, unless taken with other amino acids in the mixture. 

In fact, previous studies have highlighted the value of balancing the amino acid profile by blending isolated soy protein with dairy protein in sports nutrition [20,21,22]. Furthermore, as healthy individuals consume various types of proteins, medical diets containing multiple sources of proteins would provide a closer match to healthy normal diets than diets containing a single protein source. On a global level, plant-based foods are the leading source of protein intake, comprising 57% of daily protein intake [23]. The authors suggested that sustainably sourced plant proteins may be a promising strategy to minimize the adverse health and environmental effects of excess animal protein consumption [23]. 

A few limitations of this study need to be acknowledged. Because of the exploratory nature of the study, no multiple comparison adjustments have been performed. This should be taken into account when interpreting the outcomes of the statistical tests. Secondly, the calculation of chemical scores and the definition of the limiting amino acids for non-essential amino acids are based on a single reference, which is the first to propose such a recommendation for humans. Although these data need further investigation and validation, they are very relevant considering several reviews by experts on the importance of non-essential amino acids [3,6,9,10,11]. Moreover, it allows us to propose a new method of evaluating protein quality. Further research is needed to establish the nutritional recommendations for non-essential amino acids for human. Thirdly, we gave the protein solution (containing 18 g pure protein) as a bolus feed to fasting, healthy elderly subjects. Therefore, the results of this study most likely cannot be directly translated to patients receiving enteral tube-feed products. The proteins could be digested relatively quickly and most likely with different kinetics compared to the protein intake in complete nutrition products or via continuous tube feeding [24], or to patients in need of medical nutrition. Fourthly, we measured static concentrations of plasma amino acids and cannot determine which specific regulation process, such as digestion, absorption, splanchnic area amino acid extraction, protein synthesis, and breakdown, was responsible for the measured amino acid concentration. Stable isotope-labeled amino acid tracer studies are required to provide more insight. Finally, we were unable to measure cysteine levels, due to the limitation of the analytical techniques. Cysteine is of interest, because it is only available in low amounts in casein. Similar to other non-essential amino acids, the concentration of cysteine is reported to be lower in disease conditions, such as critical illness, and is likely to be the rate-limiting amino acid in affected patients [25]. Although cysteine can be synthesized from methionine, this metabolic conversion may be impaired, particularly during disease conditions [4,26]. 

## 5. Conclusions

Our study showed that differences in the amino acid content of protein sources were reflected in differences in post-prandial individual amino acid availability. A more balanced amino acid profile in P4 protein with higher chemical scores resulted in a more balanced post-prandial amino acid availability compared to single protein sources. Future research is required to support the hypothesized benefits of a more balanced plasma amino acid profile on whole body protein synthesis and other physiological functions.

## Figures and Tables

**Table 1:** Protein amino acid composition of the study products (gram/100gram protein).

	P4 Protein	Casein Protein	Whey Protein	Soy Protein	Pea Protein	FAO/WHO 2007 ^1^	Wu 2016 ^2^
Histidine	2.3	3.1	1.6	2.5	2.4	1.5	
Isoleucine	6.0	5.9	7.4	4.9	4.4	3.0	
Leucine	10.3	10.2	12.1	5.6	7.6	5.9	
Lysine	8.7	8.5	10.9	5.6	6.7	4.5	
Methionine	2.1	2.9	2.5	1.4	0.9	1.6	
Phenylalanine	5.0	5.5	3.8	5.5	5.7		
Threonine	5.8	4.6	8.8	3.9	3.8	2.3	
Tryptophan	1.4	1.4	1.7	1.3	0.9	0.6	
Valine	6.4	7.6	6.9	5.1	4.9	3.9	
Alanine	4.8	3.2	5.5	4.5	5.4		6.9
Arginine	5.3	3.7	2.7	7.8	8.4		7.1
Aspartic acid	11.1	7.5	12.2	11.8	11.9		6.9
Cysteine	1.5	0.4	2.7	1.2	1.0	0.6	
Glutamic acid	21.2	22.7	21.5	20.5	16.4		12.1
Glycine	3.0	1.9	2.3	4.4	4.0		7.7
Proline	6.7	10.5	6.1	4.9	4.4		8.2
Serine	6.2	6.5	6.7	5.2	5.4		4.2
Tyrosine	4.3	5.9	3.7	3.9	4.0		4.0
**Chemical score of the first and second limiting amino acid, according to FAO/WHO 2007**							
	Methionine	Cysteine	Histidine	Methionine	Methionine		
	1.31	0.67	1.05	0.84	0.56		
	Histidine	Leucine	Methione	Leucine	Valine		
	1.53	1.73	1.56	0.95	1.26		
**Chemical score of the first and second limiting amino acid, according to Wu 2016**							
	Glycine	Glycine	Glycine	Glycine	Glycine		
	0.39	0.25	0.3	0.57	0.52		
	Alanine	Alanine	Arginine	Proline	Proline		
	0.66	0.47	0.39	0.60	0.54		

^1^ WHO/FAO/UNU protein report (2007) recommendation for essential amino acids and cysteine, which is based on 0.66 g protein/kg/per day. ^2^ Exploratory calculations based on Wu (2016) recommendation for non-essential amino acids (excluding cysteine). The calculation is based on the non-essential amino acids requirements for adults >18 years at minimal physical activity and 0.66 g protein/kg per day.

**Table 2:** Subject characteristics.

Characteristics	Total (*n* = 14)
Age (years)	67.43 ± 1.5
Race	Caucasian
Gender	Female (*n* = 8), Male (*n* = 6)
Body height (m)	1.64 ± 0.10
Body weight (kg)	68.16 ± 11.38
Body mass index (kg/m^2^)	25.11 ± 2.31

All data are mean ± SD, except for gender and race.

**Table 3:** Plasma concentrations of TAA.

	P4 Protein	Casein Protein	Whey Protein	Soy Protein	Pea Protein
Baseline, μmol/L	2646.29 ± 114.08	2637.48 ± 125.26	2651.96 ± 146.36	2667.79 ± 166.52	2562.55 ± 145.35
*i*C_max_, μmol/L	1555.90 ± 351.18	1789.53 ± 314.11	1725.03 ± 419.07	1258.32 ± 322.08 *^abc^*	1255.48 ± 287.84 *^abc^*
*i*AUC, μmol/L*min	136213 ± 35888	153421 ± 35253	168149 ± 44156 *^a^*	114336 ± 27033 *^abc^*	118105 ± 35794 *^bc^*

All data are expressed as mean ± SD. *i*C_max_, maximum concentration above baseline; *i*AUC, incremental area under the curve. *^a^* indicates significant difference compared to P4 protein; *^b^* indicates significant difference compared to casein protein; *^c^* indicates significant difference compared to whey protein.

**Table 4:** Plasma concentrations of leucine, methionine, arginine, and glycine.

	P4 Protein	Casein Protein	Whey Protein	Soy Protein	Pea Protein
**Leucine**					
Baseline, μmol/L	116.13 ± 23.59	116.12 ± 19.87	115.76 ± 19.96	118/83 ± 20.37	113.92 ± 21.43
*i*C_max_, μmol/L	237.70 ± 51.22	266.38 ± 41.30	282.64 ± 80.86 *^a^*	152.46 ± 32.79 *^abc^*	165.52 ± 27.22 *^abc^*
*i*AUC,μmol/L*min	25448 ± 5647	26623 ± 5210 *^c^*	32799 ± 8505 *^a^*	16754 ± 4122 *^abc^*	18731 ± 3763 *^abc^*
**Methionine**					
Baseline, μmol/L	24.7 ± 4.23	24.59 ± 2.60	24.98 ± 2.91	24.61 ± 2.26	23.74 ± 3.53
*i*C_max_, μmol/L	24.62 ± 6.21	49.97 ± 8.24 *^ac^*	27.33 ± 7.37	12.64 ± 2.99 *^abc^*	2.88 ± 1.55 *^abc^*
*i*AUC, μmol/L*min	1936 ± 635	4360 ± 970 *^ac^*	2356 ± 794	931 ± 323 *^abc^*	137 ± 105 *^abc^*
**Arginine**					
Baseline, μmol/L	100.11 ± 17.20	101.54 ± 15.62	101.30 ± 14.68	96.13 ± 19.00	98.09 ± 12.46
*i*C_max_, μmol/L	90.96 ± 16.55	83.46 ± 13.88 *^ac^*	60.43 ± 9.58 *^a^*	102.39 ± 20.58 *^bc^*	112.45 ± 24.93 *^abc^*
*i*AUC, μmol/L*min	7979 ±1551	6534 ± 1631 *^a^*	5606 ± 1029 *^a^*	10740 ± 3257 *^abc^*	10719 ± 2426 *^abc^*
**Glycine**					
Baseline, μmol/L	281.25 ± 80.84	289.62 ± 99.50	280.54 ± 81.82	290.37 ± 82.31	273.24 ± 86.45
*i*C_max_, μmol/L	37.84 ± 21.02	34.50 ± 23.14	29.76 ±16.54	64.71 ± 43.01 *^bc^*	50.97 ± 26.2 *^bc^*
*i*AUC, μmol/L*min	1712 ± 1469	1447 ± 1147	1106 ± 735	3461 ± 2056 *^abc^*	3189 ± 2413 *^bc^*

All data are presented as mean ± SD. *i*C_max_, maximum concentration above baseline; *i*AUC, incremental area under the curve. *^a^* indicates significant difference compared to P4 protein; *^b^* indicates significant difference compared to casein protein; *^c^* indicates significant difference compared to whey protein.
